# Bacterial metabolites directly modulate farnesoid X receptor activity

**DOI:** 10.1186/s12986-015-0045-y

**Published:** 2015-11-24

**Authors:** Xianqin Zhang, Toshifumi Osaka, Satoshi Tsuneda

**Affiliations:** Department of Life Science and Medical Bioscience, Waseda University, Tokyo, 162-8480 Japan; Department of Microbiology and Immunology, Tokyo Women’s Medical University, Tokyo, 162-8666 Japan

**Keywords:** Farnesoid X receptor, Bacterial metabolites, Luciferase reporter assay, Obesity

## Abstract

**Background:**

The farnesoid X receptor (FXR), a ligand-activated transcription factor belonging to the adopted orphan receptor, plays an important role in maintaining health of the liver and intestine. In this study, we identified individual bacterial strains that directly modulated the activation of intestinal FXR.

**Methods:**

The FXR stimulatory potential of 38 bacterial strains was determined using a stable FXR reporter system derived from intestinal epithelial cells (IEC). The induction of FXR target genes by screened FXR stimulatory bacteria was determined by real-time PCR. In addition, a high fat diet (HFD)-induced obese mouse model was used to evaluate *in vivo* FXR stimulatory potential of bacterial metabolites screened in this study.

**Results:**

A luciferase assay with the FXR reporter cell line demonstrated that the FXR-stimulatory activity of most bacterial cell samples was less than 2-fold. The culture supernatants of *Bacteroides dorei* and *Eubacterium limosum* induced FXR activity and selectively regulated FXR target expression in the FXR reporter system. Treatment with *B. dorei*-derived metabolites strongly induced ileal bile acid binding protein (*IBABP*) (8.4-fold) and organic solute transporter (*OST*) α (3.1-fold) compared with *E. limosum*-derived metabolites. Furthermore, administration of *B. dorei* derived metabolites showed significant reduction in body weight gain, and both two bacterial metabolites reduced liver weight in obese mice compared to PBS-treated controls. Administration of each bacterial metabolites improved in serum levels of obesity-related metabolic biochemical markers such as ALT, AST, total cholesterol, and triglyceride. Furthermore, two bacterial metabolites enhanced the *Fxr* gene expression in the intestine and liver, and ileal *Shp* gene expression tended to be increased by treatment with the metabolites derived from *B. dorei*.

**Conclusions:**

*B. dorei* and *E. limosum* secreted the bioactive substances that directly stimulate FXR in the intestinal epithelial cells. Administration of these bacterial FXR-stimulatory metabolites improves the obesity phenotype including body weight gain, liver damage, lipid metabolism in DIO mice.

**Electronic supplementary material:**

The online version of this article (doi:10.1186/s12986-015-0045-y) contains supplementary material, which is available to authorized users.

## Background

Farnesoid X receptor (FXR, NR1H4) is a ligand-activated transcription factor belonging to the adopted orphan receptor [1]. It is abundantly expressed in the liver, intestine, kidney, and adrenals [2, 3], but is also expressed in fat, the stomach, lungs and heart [3, 4]. FXR mainly regulates intracellular levels of bile acids (BAs) in the liver and intestine though various genes directly and intervenes in other nuclear receptor signaling pathways [5, 6]. Other studies have demonstrated that FXR activates a series of genes involved in lipid and glucose homeostasis and plays a crucial role in reducing diabetes [7, 8]. Recent evidence showed that FXR activation is critical in the regulation of inflammatory responses [9–11].

FXR is a promiscuous receptor activated by many compounds not only BAs [12]. The activation of FXR by its ligands is effective for many diseases as demonstrated by in vitro and in vivo studies. FXR activation with the synthetic agonist GW4064 induced a significant reduction in hepatic and serum triglycerides levels [7, 13, 14], and very low density lipoprotein secretion was decreased by increased small heterodimer partner (*SHP*) expression in mouse models of obesity and type 2 diabetes [15]. A lower incidence and extent of necrosis, reduced inflammation, and depressed bile duct proliferation was observed in rats treated with GW4064 [16]. The FXR potent agonist 6α-ethyl-chenodeoxycholic acid (6ECDCA) protected mice from lithocholic acid-induced cholestasis [17]. In addition, 6ECDCA has anti-inflammatory effects in inflammatory bowel disease. It inhibited inflammation and preserved the intestinal barrier by inhibiting proinflammatory cytokines, such as tumor necrosis factor-α [10]. In contrast, FXR-deficiency in mice leads to increased colon cell proliferation and carcinogenesis [18], contributes to liver tumor formation [19] and causes impaired glucose tolerance and insulin sensitivity [20]. Because of its important role in BA homeostasis and other metabolic diseases, FXR has the potential to be an attractive therapeutic target for common metabolic disease treatment or prevention.

However, the clinical use of FXR ligands is not currently approved. The most potent FXR ligand is 6ECDCA, which is under investigation in a phase clinic study, and a long-term safety extension program is planned [21]. Two recent studies investigating the effect of FXR activation by GW4064 on high fat diet-induced obesity and glucose intolerance showed contrasting results [22, 23]. The underlying molecular mechanisms contributing to these differences or FXR signaling pathways are not fully understood, thus it is necessary to identify potent and selective FXR ligands or modulators that will provide us with a powerful tool to elucidate the complex mechanisms of FXR signaling.

Many types and numbers of bacteria inhabit the human body, which aid the maintenance of human metabolism homeostasis. Gut bacteria protect against obesity and insulin resistance [24], attenuate inflammation and restore colon homeostasis [25]. BAs levels were reduced in the gallbladder and small intestine in the presence of gut microbiota compared with germ free mice [26, 27]. Previous mouse studies indicated an association between gut microbiota and FXR function [28]. Gut microbiota regulate BAs homeostasis by altering the BAs composition resulting in FXR activation in the intestine and liver [29]. In addition, a recent study showed functional FXR activity was necessary for the probiotic VSL#3 to exert its activity on BAs excretion and neosynthesis in mice [30]. When investigating how gut microbiota affect relevant diseases via FXR activation, the use of individual bacterial strains is useful because of the complexity of using whole gut microbiota. Therefore, we aimed to identify individual bacterial strains from the intestine that might have important and unexpected functions via direct FXR activation.

## Methods

### Cell culture

A human colon adenocarcinoma cell line SW480 (ECACC, N0. 87092801), liver hepatocellular cell line HepG2 (ECACC, No. 85011430) and epithelial colorectal adenocarcinoma cell line CaCO-2 (ECACC, No. 86010202) were grown at 37 °C with 5 % CO_2_ in high-glucose Dulbecco’s modified Eagle’s medium (DMEM; Wako, Osaka, Japan), supplemented with 10 % heat-inactivated fetal bovine serum (Invitrogen, Grand Island, NY) and 1 % Penicillin-Streptomycin Solution (×100) (Wako).

### Preparation of bacterial suspensions and bacterial culture supernatant

Thirty-eight bacterial isolates were used in this study (Table 1). Each bacterial isolate was identified based on a nearly full 16S rRNA sequence, which was deposited in the DNA Data Bank of Japan. Bacterial strains were cultivated in GAM medium (Nissui, Tokyo, Japan) for 40 h in anaerobic conditions, and then the culture broth was centrifuged at 9000 rpm for 10 min to separate bacteria pellets and culture supernatants. After washing with 10 ml phosphate buffered saline (PBS) twice, the wet weight of bacteria pellets was measured. Then, bacteria pellets were suspended in PBS at a final concentration of 100 mg/ml as follows: PBS (ml) = W × 9, [W (g) = wet weight of bacteria pellets]. Bacterial suspensions of 0.5 ml were added to 0.2 g of 0.1 mm silica/zirconium beads (BioSpec Products, Bartlesville, OK) that were centrifuged at 5500 rpm for 2 min to disrupt bacteria. Bacterial suspensions were heat-killed at 100 °C for 10 min. Intact bacterial suspensions were directly preserved. Bacterial culture supernatant was filtered through cellulose acetate filters with a pore size of 0.2 μm (ADVANTEC Toyo, Tokyo, Japan) to remove bacteria from the supernatant. All samples were stored at −80 °C.

**Table 1:** Bacteria used in this study

Strain	Accession number	Isolation source	Taxonomic assignment	16S rRNA sequence similarity (%)
W1		Culture collections	*Lactobacillus casei* [NBRC 15883]	-
W2		Culture collections	*Lactobacillus fermentum* [NBRC 15885]	-
W3		Culture collections	*Lactobacillus plantarum* [NBRC 15891]	-
W4		Culture collections	*Lactococcus lactis* [NBRC 100933]	-
W5	LC061609	Dairy foods	*Lactobacillus gasseri* [FJ557004]	99
W6	LC061610	Dairy foods	*Lactobacillus delbrueckii* [CP000156]	99
W7	LC061611	Dairy foods	*Streptococcus thermophilus* [FR875178]	99
W8	LC061612	Dairy foods	*Lactobacillus helveticus* [CP011386]	99
W9	LC061613	Dairy foods	*Lactobacillus gasseri* [FJ557004]	99
W10	LC061614	Dairy foods	*Streptococcus thermophilus* [FR875178]	99
W11	LC061615	Dairy foods	*Lactobacillus reuteri* [EU722746]	99
W12	LC033789	Dairy foods	*Lactobacillus helveticus* [HM218413]	99
W13 (WU 12)^a^	AB932539	Human feces	*Bifidobacterium bifidum* [AP012323]	100
W14 (WU 16)^a^	AB932540	Human fces	*Bifidobacterium longum* [FP929034]	100
W15 (WU 22)^a^	AB932542	Human feces	*Bifidobacterium adolescentis* [CP010437]	99
W16 (WU 57)^a^	AB932544	Human feces	*Bifidobacterium bifidum* [KJ160509]	99
W18	LC033790	Human feces	*Bacteroides dorei* [EU722737]	99
W19	LC033791	Human feces	*Eubacterium limosum* [AB638446]	99
W20	LC033792	Human feces	*Bacteroides* sp.W20 [EU728710]	99
W21	LC033793	Human feces	*Bacteroides fragilis* [AB618792]	98
W22	LC033794	Human feces	*Ruminococcus* sp.W22 [FJ611794]	99
W23	LC033795	Human feces	*Clostridiales bacterium* W23 [HQ452859]	98
W24	LC033796	Human feces	*Bacteroides uniformis* [AB247142]	99
W25	LC033797	Mouse feces	*Parabacteroides distasonis* [AB238924]	98
W26	LC033798	Mouse feces	*Bacteroides acidifaciens* [AB510696]	97
W27	LC033799	Mouse feces	*Bacteroides thetaiotaomicron* [AE015928]	97
W28	LC033800	Mouse feces	*Lactobacillus johnsonii* [FN298497]	99
W29	LC033801	Mouse feces	*Lactobacillus reuteri* [KR492886]	97
W30	LC033802	Mouse feces	*Lactobacillus animalis* [AB911535]	98
W31	LC033803	Mouse feces	*Bacteroides sartorii* [AB572597]	98
W32	LC033804	Mouse feces	*Bacteroides* sp.W32 [AB599946]	99
W33	LC033805	Mouse feces	*Parabacteroides goldsteinii* [AB547650]	99
W34	LC033806	Mouse feces	*Enterococcus faecalis* [FJ378702]	99
W35	LC033807	Human feces	*Enterococcus durans* [AJ276354]	99
W36 (WU 27)^a^	AB932524	Human feces	*Enterococcus raffinosus* [AF061003]	99
W37 (WU 65)^a^	AB932534	Human feces	*Enterococcus cecorum* [AF061009]	99
W38 (WU 76)^a^	AB932546	Human feces	*Enterococcus avium* [DQ779961]	100
W39	LC033808	Human feces	*Enterococcus faecium* [FJ378690]	99

^a^These strains are the same as those previously reported [44]

### FXRE-driven firefly luciferase reporter vector

A DNA fragment containing four copies of the FXR element (FXRE: 5′-aaactgaGGGTCAgTGACCCaaggtgaa-3′) from the phospholipid transfer protein promoter [31, 32] and *Xho*I and *Bgl*II restriction enzyme sites was synthesized and cloned into the vector pUC19 (Greiner Bio-One, Frickenhausen, Germany). The *Xho*I/*Bgl*II fragment of pUC19-4 × FXRE was ligated into a pGL4.27 [luc2p/minP/Hygro] vector (Promega, Madison, WI) and digested with *Xho*I/*Bgl*II (Promega) to generate a FXRE-driven firefly luciferase reporter vector (pGL4-4 × FXRE-*luc*).

### Establishment of a stable FXR reporter cell line

SW480 cells do not respond to treatment with a FXR agonist (e.g. GW4064), because SW480 cells do not endogenously express FXR [33]. To generate a stable FXR expressing cell, SW480 cells were transfected with a FXR expression vector EX-T0601-M02 (Genecopeia, Rockville, MD) using FuGENE reagent (Promega). For the selection of stable FXR expressing cells, cells were cultured in DMEM medium containing 800 μg/ml G418 (Wako). G418-resistant SW480 cells were further transfected with a reporter vector pGL4-4 × FXRE-*luc* with FuGENE transfect reagent. For the selection of a stable FXR reporter vector cell, the cells were cultured in DMEM medium containing 800 μg/ml G418 and 300 μg/ml hygromycin B (Invitrogen). G418 and hygromycin B-resistant cells were collected with a cloning cylinder, and subcultured into fresh medium containing G418 and hygromycin B.

### Reporter assay

FXR reporter cells were seeded at a density of 5 × 10^4^ cells/well (96-well plates). Twenty-four hours after seeding, cells were incubated with bacterial suspensions, culture supernatants, dimethyl sulfoxide (DMSO, 0.1 % v/v) or GW4064 (10 μM) respectively for 24 h. Then, cell supernatants were removed and cells were washed twice with phosphate-buffered saline (PBS) and lysed by adding 20 μl of passive lysis 5 × buffer (Promega) with gentle rocking for 20 min. Luciferase activity was measured by the administration of luciferase assay reagent (Promega) using a GloMax® 96 Microplate Luminometer (Promega). The ratio of treatment over control was used to determine the fold activation.

### RNA isolation and Real-time PCR

Cells (3 × 10^5^ cells/well) were seeded in a 24-well plate 24 h before the administration of bacterial supernatant samples. After 24 h of incubation with bacterial supernatant samples, total RNA was extracted from cells using a Qiagen RNeasy mini kit (Qiagen, Tokyo, Japan). RNA concentration and purity were determined by a NanoDrop™ spectrophotometer (Thermo Scientific, Waltham, MA). For reverse transcription reactions, 1 μg of total RNA was used in a final volume of 20 μl with PrimeScript RT reagent Kit (Takara, Tokyo, Japan) at 37 °C for 15 min and 85 °C for 5 s. Real-time PCR reactions were performed using the iCycler iQ™ Real-Time PCR Detection System (Bio-Rad, Hercules, CA). For each reaction, the final volume of 20 μl contained 10 μl of SYBR Green PCR Mix (Bio-Rad), 1 μl of each primer (10 μM) (Table 2), and 2 μl of RT product diluted 10 times. After PCR, melting curve analysis was performed to ensure the specificity of the assay. Each analysis was performed in triplicate and glyceraldehyde-3-phosphate dehydrogenase (GAPDH) was used as endogenous gene. Relative gene expression was calculated via the Relative standard curve method [34].

**Table 2:** Sequences of primers used in this study

Gene	Forward	Reverse	Reference
*GAPDH*	GAAGGTGAAGGTCGGAGT	CATGGGTGGAATCATATTGGAA	[45]
*IBABP*	TCACTTGGTCCCAGCACTA	CTTGTCACCCACGATCTCT	[45]
*OSTα*	CTACACCTGGGTGAGCAGAA	AGAGGAATAGGGAGGCGAAC	[46]
*FGF19*	CACGGGCTCTCCAGCTGCTTCCTGCG	TCCTCCTCGAAAGCACAGTCTTCCTCCG	[47]
*SHP*	GGCTGGCAGTGCTGATTCAG	TGGGGTGTGGCTGAGTGAAG	[48]
*Gapdh*	AGGTCGGTGTGAACGGATTTG	TGTAGACCATGTAGTTGAGGTCA	[22]
*Fxr*	TCCAGGGTTTCAGACACTGG	GCCGAACGAAGAAACATGG	[27]
*Ibabp*	CAGGAGACGTGATTGAAAGGG	GCCCCCAGAGTAAGACTGGG	[27]
*Ostα*	TGTTCCAGGTGCTTGTCATCC	CCACTGTTAGCCAAGATGGAGAA	[27]
*Fgf15*	ACGTCCTTGATGGCAATCG	GAGGACCAAAACGAACGAAAT T	[27]
*Cyp7a1*	AGCAACTAAACAACCTGCCAGTACTA	GTCCGGATATTCAAGGATGCA	[27]
*Cyp7b1*	TAGCCCTCTTTCCTCCACTCATA	GAACCGATCGAACCTAAATTCCT	[27]
*Cyp8b1*	GGCTGGCTTCCTGAGCTTATT	ACTTCCTGAACAGCTCATCGG	[27]
*Shp*	CGATCCTCTTCAACCCAGATG	AGGGCTCCAAGACTTCACACA	[27]
*Bsep*	CTGCCAAGGATGCTAATGCA	CGATGGCTACCCTTTGCTTCT	[27]
*Ntcp*	ATGACCACCTGCTCCAGCTT	GCCTTTGTAGGGCACCTTGT	[27]
*Ibat*	ACCACTTGCTCCACACTGCTT	CGTTCCTGAGTCAACCCACAT	[27]

Genes in capitals indicate human genes, and those in lower case are mouse genes. *Gapdh, Ibabp* and *Ostα* were analyzed using the following conditions: 95 °C 2 min, 40 cycles of 95 °C for 10 s, and 60 °C for 30 s. *Fgf19* was analyzed using the following conditions: 95 °C 15 min, 40 cycles of 95 °C for 15 s, and 62 °C for 30 s. All mouse genes were analyzed using the following conditions: 95 °C 2 min, 40 cycles of 95 °C for 15 s, and 60 °C for 60 s

### In vivo study

Four-week-old C57BL/6 J male mice were obtained from CLEA Japan. Mice were housed in a temperature-controlled room (23 °C) under a 12 h light–dark cycle. The mice were given standard diet (STD; CLEA Japan, Tokyo, Japan) for 1 week before high fat diet (HFD; CLEA Japan) administration for another 10 weeks. Mice were also administered bacterial culture supernatants (0.1 ml) per day by intragastric administration. Body weight was recorded and HFD intake was estimated weekly. Mice were sacrificed after 11 weeks of bacterial culture supernatants intervention. Tissues were collected, snap-frozen in liquid nitrogen and stored at −80 °C until analyzed. Total RNA was isolated from the ileum and liver using a Qiagen RNeasy mini kit after homogenization (Microtec, Chiba, Japan). Serum levels of aspartate aminotransferase (AST), alanine aminotransferase (ALT), total cholesterol, glucose, triglyceride were determined with a SPOTCHEM™ EZ SP-4420 analyzer (Arkray, Tokyo, Japan). All experiments were approved by the Waseda University Academic Research Ethics Committee.

## Results

### Stable FXR reporter cell line construction

**Fig. 1:**
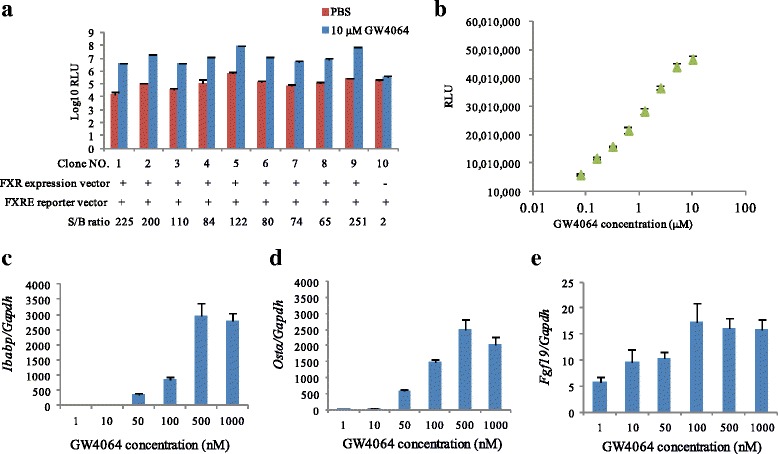
Construction of a stable FXR reporter cell line. Cells were seeded to white 96-well plates with density of 5 × 10^4^/well 24 h before administration of GW4064 or DMSO. After 24 h cultivation, cells were lysed and chemiluminescense was evaluated by administration of luciferase assay reagent: (**a**) RLU and S/B ratio. (**b**) Dose response of FXR agonist (GW4064). (**c**-**e**) mRNA expression of FXR target genes, *Ibabp* (**c**), *Ostα* (**d**), *Fgf19* (**e**) by GW4064. *Gapdh* was used as endogenous gene. Gene expression levels were calculated via the relative standard curve method. Experiments were performed in triplicate with the mean ± SD shown. RLU: Relative luminescent units; S/B: Signal noise ratio = GW4064/DMSO = Relative FXR stimulatory potential

To monitor FXR transcriptional activity in an intestinal epithelial cell line, we constructed a stable FXR reporter cell line by cotransfecting a reporter vector pGL4-4xFXRE-*luc* and human FXR expression vector into SW480 cells. Nine single cell clones isolated from the transfected cell populations were used to determine the responsiveness to synthetic FXR agonist (GW4064). Each FXR reporter cell clone was treated with 10 μM GW4064 or DMSO as a control. Clone 9 was selected because it had a high relative luminescent unit (RLU) and signal-to-background (S/B) ratio (Fig. 1a). The responsiveness of SW480 cells transfected with the reporter vector pGL4-4xFXRE-*luc* to 10 μM GW4064 was very low (S/B ratio = 2), indicating that endogenous FXR expression level in SW480 cells is low. Thus, agonist-induced luciferase induction in FXR reporter cells constructed in this study was due to exogenous FXR expression.

Next, FXR reporter cells were exposed to different concentrations of FXR agonist GW4064 to characterize agonist dose-responses (Fig. 1b). The level of FXR reporter activity increased in a dose-dependent manner. Furthermore, we determined whether stimulation with FXR agonist could transactivate FXR target genes in FXR reporter cells. GW4064 induced the gene expression of ileal bile acid binding protein (*Ibabp*), organic solute transporter α (*Ostα*) and fibroblast growth factor 19 (*Fgf19*) in a dose-dependent manner (Figs. 1c-e). The mRNA levels of the gene *Ibabp* and *Ostα* reached a maximum expression level at 500 nM GW4064, while the expression of the *Fgf19* gene reached a peak of expression at 100 nM GW4064.

### Screening of FXR-stimulating bacteria by luciferase assay

**Fig. 2:**
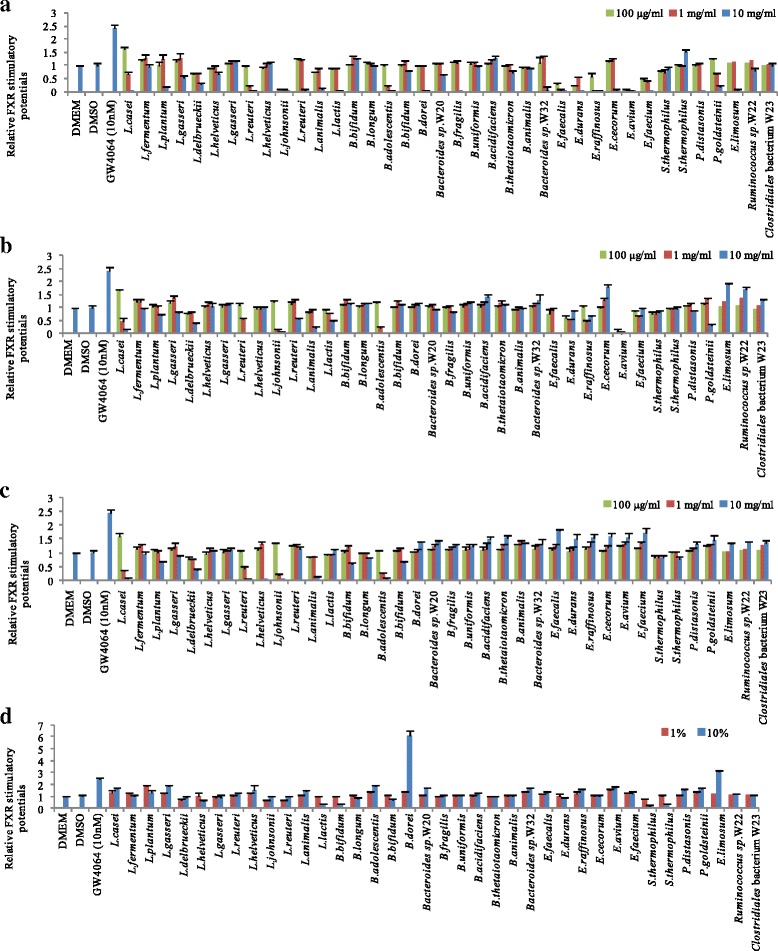
Screening of FXR-stimulating bacteria. After cells were cultured in white 96-well plates with density of 5 × 10^4^/well for 24 h, bacterial suspensions or culture supernatants were introduced for 24 h incubation before measurement of chemiluminescense by administration of luciferase assay reagent. (**a**) Intact bacteria. (**b**) Mechanical disrupted bacteria. (**c**) Heat-killed bacteria. (**d**) Culture supernatants. Experiments were performed in triplicate. Values are the mean ± SD

By using a FXR reporter cell (clone 9), a total of 38 bacterial strains, which were affiliated with the genera *Bacteroides*, *Bifidobacterium, Enterococcus*, *Eubacterium*, *Lactobacillus*, *Parabacteroides*, *Ruminococcus* and *Streptococcus*, were assessed to determine whether they could modulate FXR activation (Fig. 2). FXR-stimulatory potentials of intact bacterial cells, mechanical disrupted bacterial cells, heat-killed bacterial cells, or bacterial culture supernatants of each isolate were evaluated using FXR reporter cells. Intact bacteria maintained the integral part of the bacterial outer membrane, whereas fractionated subcellular components were exposed when bacteria were disrupted by beating with beads. Heat treatment of bacterial cells leads to conformational changes and the degradation of bacterial cell wall components. FXR-stimulatory activities of most bacterial cell samples showed less than 2-fold changes. Interestingly, culture supernatants derived from *B. dorei* and *E. limosum* intensely activated FXR, which indicated that these two bacteria might function as FXR modulators (Fig. 2d).

### Evaluation of FXR-stimulating bacteria

**Fig. 3:**
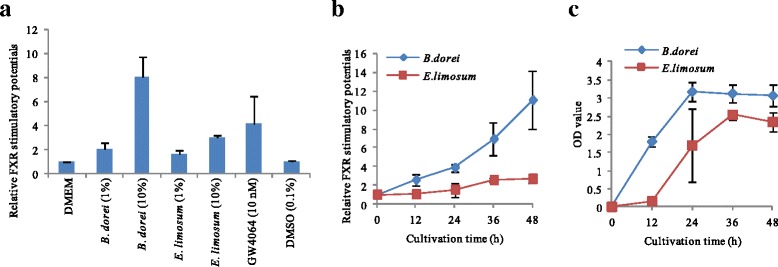
Evaluation of two FXR-stimulating bacteria. After cells were cultured in white 96-well plates with density of 5 × 10^4^/well for 24 h, bacterial culture supernatants were introduced for 24 h incubation before measurement of chemiluminescense by administration of luciferase assay reagent: (**a**) FXR activation by culture supernatants of *B. dorei* and *E. limosum*, (**b**) FXR activation by culture supernatants sampled at various times, (**c**) The quantity of two bacterial cells at different times. Values are the mean ± SD (*n* = 3). *p < 0.05 compared to the GAM group

By repeating three independent experiments in triplicate, we confirmed the reproducibility of the FXR-stimulatory activity of culture supernatants derived from *B. dorei* and *E. limosum* (Fig. 3a). As a result, 10 % of *B. dorei* culture supernatant strongly induced FXR activation, whereas the FXR-stimulatory potential of *E. limosum* was similar to that of 10 nM GW4064. Next, the FXR-stimulatory activities of bacterial culture supernatants were sampled every 12 h and bacterial cell growth during cultivation was determined (Figs. 3b, c). The FXR-stimulatory potential of *B. dorei* in culture supernatant increased continuously with the growth of bacterial cells. In contrast, the FXR-stimulatory potential of *E. limosum* in the culture supernatant began to increase after 12 h and reached a plateau at 36 h.

### Induction of FXR target genes by bacterial culture supernatants

**Fig. 4:**
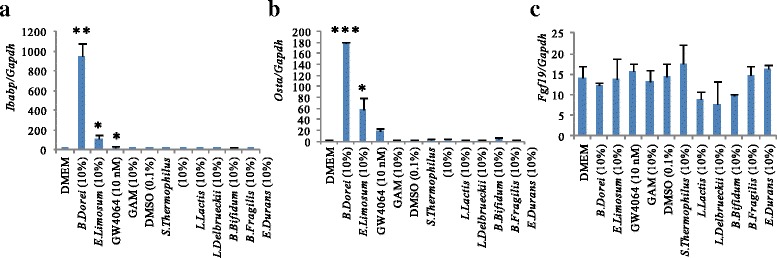
mRNA expression of FXR target genes induced by *B. dorei* or *E. limosum.* Cells were cultured in 24-well plates with density of 3 × 10^5^/well for 24 h, bacterial culture supernatants were introduced for 24 h incubation before total RNA isolation. mRNA levels were normalized to *Gapdh* mRNA levels via the relative standard curve method: (**a**) *Ibabp* expression levels, (**b**) *Ostα* expression levels, (**c**) *Fgf19* expression levels. Values are the mean ± SD (*n* = 3). Differences compared with the GAM culture treatment group were calculated using Student’s *t*-test (*p < 0.05, **p < 0.01, ***p < 0.001)

We determined whether the culture supernatants of *B. dorei* and *E. limosum* induced FXR target gene expression in FXR reporter cells. As shown in Fig. 4, culture supernatants derived from both *B. dorei* and *E. limosum* transactivated the FXR target genes *Ibabp* and *Ostα*. Interestingly, treatment with *B. dorei*-derived metabolites strongly induced *Ibabp* mRNA (8.4-fold) and *Ostα* (3.1-fold) compared with *E. limosum*-derived metabolites. Nevertheless, neither of these two culture supernatants induced *Fgf19* mRNA expression in FXR reporter cells. These results indicated that FXR activation induced by treatment with these bacterial metabolites did not induce the transactivation of all FXR target genes.

### Specificity of FXR regulation by two bacterial metabolites

**Fig. 5:**
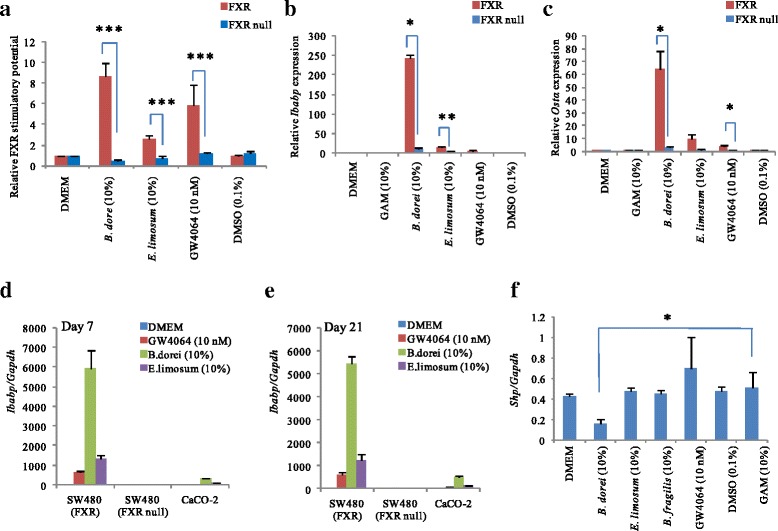
Intestinal epithelial cell-specific FXR activation of two bacterial metabolites. FXR-expressed or FXR-null SW480 cells were treated with bacterial culture supernatants (10 % v/v) for 24 h incubation before measurement of by administration of luciferase assay reagent. (**a**) The activation of FXR in each cell was measured by luciferase reporter construct FXRE-Luc (*n* = 2). The induction of FXR target gene by bacterial culture supernatants was determined with quantitative real-time reverse transcription-PCR analysis (*n* = 1): (**b**) *Ibabp* gene, (**c**) *Ostα* gene. (**d**, **e**) The culture supernatant derived from *B. dorei* transactivated FXR target gene (*Ibabp*) in Caco-2 cells (*n* = 1). Before the treatment with bacterial supernatants, cells were cultured for 7 days or 21 days. The induction of FXR target gene (*Ibabp*) by bacterial culture supernatant were determined in undifferentiated (**d**) or fully differentiated Caco-2 cells (**e**). (**f**) The FXR stimulatory potential of bacterial supernatants FXR in HepG2 cells. *Shp* gene expression levels in HepG2 cells treated with bacterial culture supernatant were determined (*n* = 1). mRNA levels were normalized to *Gapdh* mRNA levels via the relative standard curve method. Relative mRNA expression: Compared to DMEM medium group. Experiments were performed in triplicate. Values are the mean ± SD. Differences were calculated using Student’s *t*-test (*p < 0.05, **p < 0.01, ***p < 0.001)

Due to multiple possibilities of luciferase activity induction, the specificity of FXR activation by two bacterial metabolites was validated in cells with or without FXR expression. As shown in Fig. 5a, two bacterial metabolites did not induce FXR activity in FXR null SW480 cells, indicating that the chemiluminescense activity stimulated by two bacterial metabolites is dependent on FXR. In addition, the levels of FXR target gene *Ibabp* and *Ostα* were very low compared with that in FXR containing cells (Figs. 5b, c), indicating that FXR target genes expression by two bacterial culture supernatants is dependent on FXR in SW480 cells.

To investigate whether two bacterial metabolites activation FXR target genes in other cell lines, we used two different cell lines (i.e. Caco-2, HepG2), which endogenously express FXR [33, 35, 36]. Two bacterial culture supernatants induced the *Ibabp* gene expression (Figs. 5d, e). The levels of *Ibabp* gene expression were consistent with differentiation degree of Caco-2 cells. However, two bacterial metabolites did not induce the *Ibabp* gene expression in FXR null-SW480 cells. Also, the culture supernatant derived from *B. dorei* did not stimulate FXR of a hepatocyte-derived cell line, HepG2 cells, by measuring the *Shp* gene that induced directly by FXR activation (Fig. 5f).

### Beneficial effects of daily administration of bacterial metabolites in diet-induced obese (DIO) mice

**Fig. 6:**
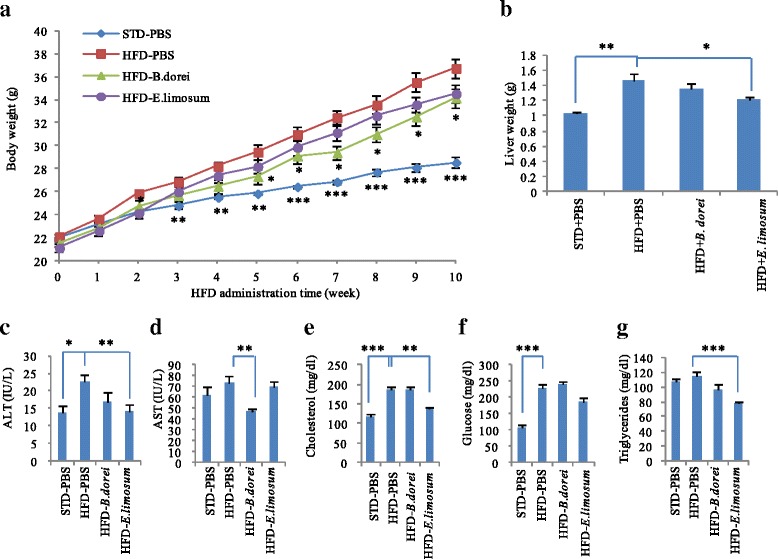
Effects of FXR-stimulatory metabolites on the phenotypes in diet-induced obesity mice. Mice fed with high fat diet (HFD) were treated bacterial culture supernatants (0.1 ml) by intragastric administration. (**a**) Body weight changes during the experimental period. (**b**) Liver weight changes after 10 weeks of the treatment. (**c**-**g**) Serum biochemical markers. Values are the mean ± SEM (standard diet-fed group, *N* = 3; HFD-fed group, *N* = 6). Differences compared to HFD-PBS group were calculated using Student’s *t*-test (*p < 0.05, **p < 0.01, ***p < 0.001)

To investigate whether *B. dorei* and *E. limosum* FXR-derived metabolites conferred anti-obesity effects, they were daily administrated to diet-induced obese mice fed with high-fat diet (HFD). Mice body weight recorded every week is shown in Fig. 6a. HFD dramatically elevated mice body weight compared to STD. Since 6 weeks of administration of *B. dorei* derived-metabolites (the period of HFD intake, 5 weeks), the mice showed lower body weight compared with mice that received PBS only, indicating that *B. dorei* culture metabolites may help mice to be resistant to the body weight gain. In contrast, the mice that received those of *E. limosum* did not show any significant differences in body weight gain. Long-term administration of each bacterial metabolite had a reducing effect of liver weight in DIO mice (Fig. 6b). Especially, administration of *E. limosum*-derived metabolites led to a significant reduction in liver weight compared with the PBS control group. On the other hand, DIO mice treated with *B. dorei*-derived metabolites tended to reduce liver weight compared with the PBS control group, but this was not statistically significant.

Daily administration of each bacterial metabolites had improvement in serum biochemical markers of liver functions (Figs. 6c-g). Compared to PBS-treated DIO mice, AST levels decreased significantly in mice treated with metabolites derived from *B. dorei*. Furthermore, *E. limosum*-derived metabolites have a great improvement in serum levels of ALT, total cholesterol, and triglyceride. These results suggest that FXR-stimulatory bacterial metabolites can improve the metabolic conditions in DIO mice.

We also determined whether alive bacterial cells of two FXR-stimulatory bacteria promote the metabolic improvement in DIO mice. However, the mice treated with alive bacterial cells per week were significantly indistinguishable from the PBS-treated mice in regard to body weight gain, liver weight, serum biomarkers (Additional file 1: Figure S1).

### Bacterial metabolite-induced modulation of FXR target genes

**Fig. 7:**
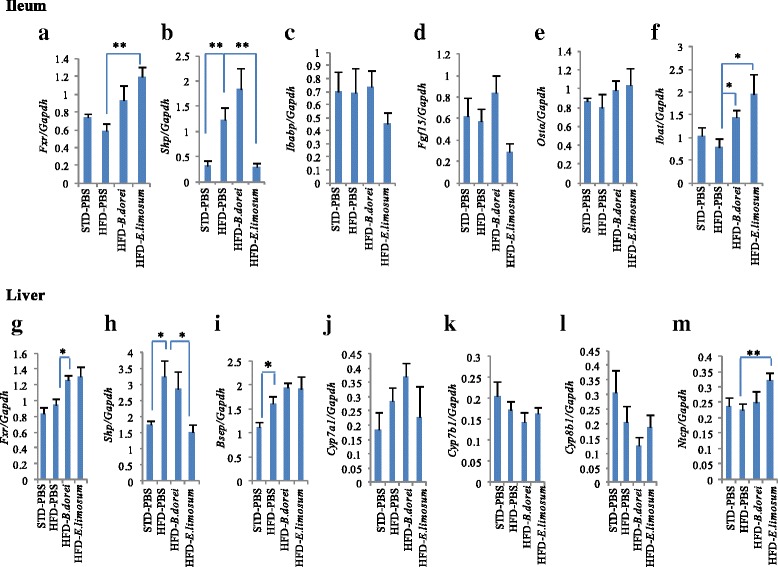
Effects of FXR-stimulatory metabolites on intestinal and hepatic expression of FXR target genes. (**a-f**) mRNA levels in the ileum. (**g-m**) mRNA levels in the liver. mRNA levels were normalized to *Gapdh* mRNA levels via the relative standard curve method. Values are the mean ± SEM (*N* = 6). Differences from the PBS treatment group were calculated using Student’s *t*-test (*p < 0.05, **p < 0.01)

To determine whether the administration of FXR-stimulatory bacteria affected the regulation of FXR target genes in vivo, we measured mRNA expression levels of *Fxr* target genes in the ileum and liver (Fig. 7). The HFD intake did not affect ileal or hepatic *Fxr* expression levels, while enhanced the expression levels of ileal *Shp* (3.7-fold), hepatic *Shp* (1.8-fold) and *Bsep* (1.4-fold) compared with PBS-treated DIO mice. Administration of two bacterial metabolites induced *Fxr* expression in both ileum and liver (Figs. 7a, g). *E. limosum-*derived metabolites significantly up-regulated the expression of ileal bile acid transporter (*Ibat*, 2.5-fold) and Na^+^-taurocholate cotransporting polypeptide (*Ntcp*, 1.4-fold), but decreased the expression of ileal *Shp* (0.2-fold) and hepatic *Shp* (0.5-fold). *B. dorei*-derived metabolites significantly increased the ileal *Ibat* expression (1.9-fold). These results indicate that two different bacterial metabolites can modulate the expression of FXR target genes in a tissue- and gene-specific manner.

We also determined whether administration of alive bacterial cells of *B. dorei* and *E. limosum* alter the expression of FXR target genes in DIO mice (Additional file 2: Figure S2). As a result, the administration of alive bacterial cells enhanced ileal *Fxr* mRNA expression, while had no effect on other FXR target genes in the ileum and liver.

## Discussion

In the present study, we investigated whether bacteria or bacterial metabolites could modulate FXR activation. Our results indicated that culture supernatants of *B. dorei* and *E. limosum* induced FXR activity and selectively induced the expression of FXR target genes in IEC. We also found that the administration of culture supernatants of *B. dorei* and *E. limosum* to DIO mice increased *Fxr* activity and selectively regulated the expression of *Fxr* target genes in both ileum and liver. The present study showed for the first time that bacterial culture supernatants directly induce FXR activity and the expression of FXR target genes.

Cell-based reporter assays are widely used for the screening of FXR agonists or antagonists isolated from natural or synthetic compounds. Most FXR reporter cells used in previous studies were derived from hepatocyte-derived cell line (HepG2) or human embryonic kidney cell line (HEK293), but not IECs [35, 36]. In the present study, an IEC-based FXR reporter system was developed for the identification of gut bacteria that modulate intestinal FXR signaling. The expression and function of FXR in IECs are important factors for the construction of an IEC-based FXR reporter system. Gottardi et al. conducted a comparative evaluation on the level of FXR expression among commonly-used human colonic carcinoma cell lines (Caco-2, HT-29, SW480, SW620) and reported the dependency of FXR expression levels on cellular differentiation [33]. In this report, the level of FXR expression in Caco-2 and HT-29 cells changed with their degree of differentiation, which might affect the repeatability and reproducibility of the assay system. Although SW480 or SW620 cells did not endogenously express FXR, when these cell lines were transiently transfected with the expression vector for FXR, they could normally transactivate FXR target genes by stimulation with a FXR agonist. Thus, the stable transfection of SW480 cells with an expression vector for human FXR and a FXRE-luciferase reporter construct is important for IEC-based FXR reporter systems with high reproducibility and superior signal-to-background ratio. Furthermore, two bacterial metabolites did not induce chemiluminescense activity in SW480 cells without FXR expression, indicating that two bacterial metabolites contain a FXR agonist. In addition, *B. dorei* and *E. limosum* screened using IEC-based FXR reporter cells induced FXR target gene *Ibabp* expression in Caco-2 cells but did not activate FXR signal transduction in a HepG2 cell line that expresses FXR endogenously with normal functions [37]. These results indicate that IEC-based FXR reporter cell is useful for screening intestinal FXR modulators including bacteria.

FXR activation in the intestine and liver directly induces the expression of genes regulating the transport of bile acids (BAs) (e.g. *Bsep*, *IBABP*, *OSTα*), while the genes involved in the synthesis (e.g. *Cyp7a1*, *Cyp8b1*) and re-absorption (e.g. *Ibat*, *Ntcp*) of BAs are repressed through SHP and FGF15/19 induced by FXR activation [38]. Two bacterial metabolites enhanced the *Fxr* gene expression in the intestine and liver, and ileal *Shp* gene expression tended to be increased by treatment with the metabolites derived from *B. dorei*. However, the expression of most FXR target genes were not positively regulated by intragastric administration of the bacterial supernatants. Rather, *E. limosum*-derived metabolites significantly repressed ileal and hepatic expression of *Shp* and up-regulated the expression of *Ibat* and *Ntcp*. Furthermore, the administration of alive bacterial cells had little influence on the expression of the FXR target genes. The difference between in vitro and in vivo experiments might be affected by the colonization and metabolic activities of FXR-stimulatory bacteria in the ileum. Previous studies showed that the composition of BAs, which are strongly affected by gut microbiota, modulated FXR-mediated gene expression in vivo [27, 28, 30, 39]. Thus, the alteration in gut microbiota by FXR-stimulating bacterial metabolites might also influence the regulation of FXR target gene expression. In future study, we need to investigate whether BAs metabolism and gut microbiota are influence by the administration of FXR-stimulatory bacteria or metabolites.

Intestinal FXR signaling might be a drug target for obesity and metabolic complications (e.g. non-alcoholic fatty liver disease). In this study, HFD-fed mice treated with FXR-stimulating bacterial metabolites (*B. dorei*) helped mice to be resistant to obesity compared with control mice. Furthermore, in vivo administration of FXR-stimulating bacterial metabolites decreased the levels of serum biochemical markers for liver injury (i.e. ALT, AST) and lipid metabolism (i.e. cholesterol, triglyceride) in DIO mice. At present, there are contradictory reports on the role of intestinal FXR signaling in metabolic improvement. One potential mechanism is that the inhibition of intestinal FXR signaling improves obesity and insulin responsiveness in HFD-fed mice [28]. Previous reports showed that diet-induced weight gain or metabolic defects were suppressed in intestine-specific FXR-null mice [28]. It was also reported that the anti-obesity effect was associated with the inhibition of intestinal FXR signaling by the accumulation of tauro-β-muricholic acid (TβMCA), which is an endogenous antagonist of FXR [27]. The accumulation of TβMCA is caused by the depletion or decrease of gut microbiota that possess BAs deconjugation ability. However, intestinal FXR activation with the FXR agonist fexaramine enhanced energy expenditure mediated through the activation of β-adrenergic receptor signaling in adipose tissues, resulting in metabolic improvement in DIO mice [39]. The intestinal-selective effect of FXR might be coordinated by the induction of intestinal endocrine hormone fibroblast growth factor 15 (*FGF15*, homolog of *FGF19* in humans) [27, 30, 39–42]. We showed that the administration of bacterial cells or metabolites of *B. dorei* tended to enhance the expression of ileal *Fgf15* gene compared with the PBS-treated group although this was not statistically significant. This suggests that the concentration of FXR agonist in the supernatants or the frequency of bacteria might be inadequate to achieve metabolic improvement in DIO mice. Further studies are required to evaluate whether metabolic improvement is enhanced by the increased administration frequency of bacterial cells or administration of FXR agonists purified from bacterial culture supernatants.

Intestinal FXR activation has the potential of curing intestinal bowel disease (IBD) as well as metabolic disorders [9, 10]. A previous report showed that intestinal inflammation was associated with a decrease *Fxr* expression levels in the inflamed intestinal mucosa of Crohn’s disease and experimental colitis mice [9]. Polymorphisms in the FXR gene were not associated with IBD pathogenesis [43]. Thus, intestinal FXR activation could have a therapeutic effect on IBD. Interestingly, the expression level of *Fxr* mRNA in the ileum was enhanced by treatment with both FXR-stimulating bacteria screened in this study. In future experiments, we will evaluate whether *B. dorei* or *E. limosum* exerts a curative effect on intestinal inflammation using experimental colitis mouse models.

In summary, this study has been successful in screening two distinct gut bacteria secreted metabolites that directly transactivated FXR target genes in IEC by using a cell-based assay. Long-term administration of *B. dorei*-derived metabolites, which have high FXR-stimulatory potential in vitro, suppress the obese phenotype including weight gain and liver damage in DIO mice. However, further studies are required to validate the effects and safety of FXR-stimulatory bacteria or metabolites as a therapeutic option for metabolic modulation in animal models.

## Conclusions

In this study, we report that metabolites of *B. dorei* and *E. limosum* induced FXR activity in a stable FXR reporter system, and selectively regulated FXR target gene expression in vitro. Treatment of diet-induced obese mice with *B. dorei* or *E. limosum* metabolites regulated the expression of genes involved in BAs homeostasis in a gene- and tissue-specific manner. Thus, these two bacteria have potential value as useful tools for the study of complex FXR functions, and provide a novel therapeutic direction for the treatment or prevention of common metabolic disorders.

## Additional files

Additional file 1: Figure S1. Effects of FXR-stimulatory bacteria on the phenotypes in diet-induced obesity mice. HFD-fed Mice fed with high fat diet (HFD) were treated with alive bacterial cells (109 bacteria) by intragastric administration. (A) Body weight changes during the experimental period. (B) Liver weight changes after 10 weeks of the treatment. (C-G) Serum biochemical markers. Values are the mean ± SEM (standard diet-fed group, *N* = 3; HFD-fed group, *N* = 6). Differences compared to HFD-PBS group were calculated using Student’s *t*-test (**p* < 0.05, ***p* < 0.01, ****p* < 0.001). (PPTX 75 kb) Additional file 2: Figure S2. Effects of FXR-stimulatory bacteria on intestinal and hepatic expression of FXR target genes. (A-F) mRNA levels in the ileum. (B) mRNA levels in the liver. mRNA levels were normalized to *Gapdh* mRNA levels via the relative standard curve method. Values are the mean ± SEM, *n* = 6. Differences from the PBS treatment group were calculated using Student’s *t*-test (**p* < 0.05, ***p* < 0.01). (PPTX 108 kb)

## Abbreviations

FXR: Farnesoid X receptor
Shp: Small heterodimer partner
6ECDCA: 6α-ethyl-chenodeoxycholic acid
BAs: Bile acids
FXRE: FXR element
DMSO: Dimethyl sulfoxide
PBS: Phosphate-buffered saline
Gapdh: Glyceraldehyde-3-phosphate dehydrogenase
RLU: Relative luminescent unit
MCAs: Muricholic acids
Ibabp: Ileal bile acid binding protein
Ostα: Organic solute transporter α
Fgf19: Fibroblast growth factor 19
Fgf15: Fibroblast growth factor 15
HFD: High fat diet
Ibat: Ileal bile acid transport
Bsep: Bile salt export pump
Cyp8b1: Sterol 12-α-hydroxylase
Cyp7b1: 25-hydroxycholesterol 7-α-hydroxylase
Cyp7a1: Cholesterol 7-α-hydroxylase
Ntcp: Na+/taurocholate cotransporting polypeptide
IEC: Intestinal epithelial cells
TβMCA: Tauro-β-muricholic acid
IBD: Intestinal bowel disease
