# Supplementary material for: Bacterial metabolites directly modulate farnesoid X receptor activity
Source: Nutr Metab (Lond). 2015 Nov 24;12:48. doi: 10.1186/s12986-015-0045-y (PMC4657204; doi:10.1186/s12986-015-0045-y)
Supplement: Additional file 1: Figure S1. — Effects of FXR-stimulatory bacteria on the phenotypes in diet-induced obesity mice. HFD-fed Mice fed with high fat diet (HFD) were treated with alive bacterial cells (109 bacteria) by intragastric administration. (A) Body weight changes during the experimental period. (B) Liver weight changes after 10 weeks of the treatment. (C-G) Serum biochemical markers. Values are the mean ± SEM (standard diet-fed group, N = 3; HFD-fed group, N = 6). Differences compared to HFD-PBS group were calculated using Student’s t-test (*p < 0.05, **p < 0.01, ***p < 0.001). (PPTX 75 kb) [file 12986_2015_45_MOESM1_ESM.pptx]

## Slide 1
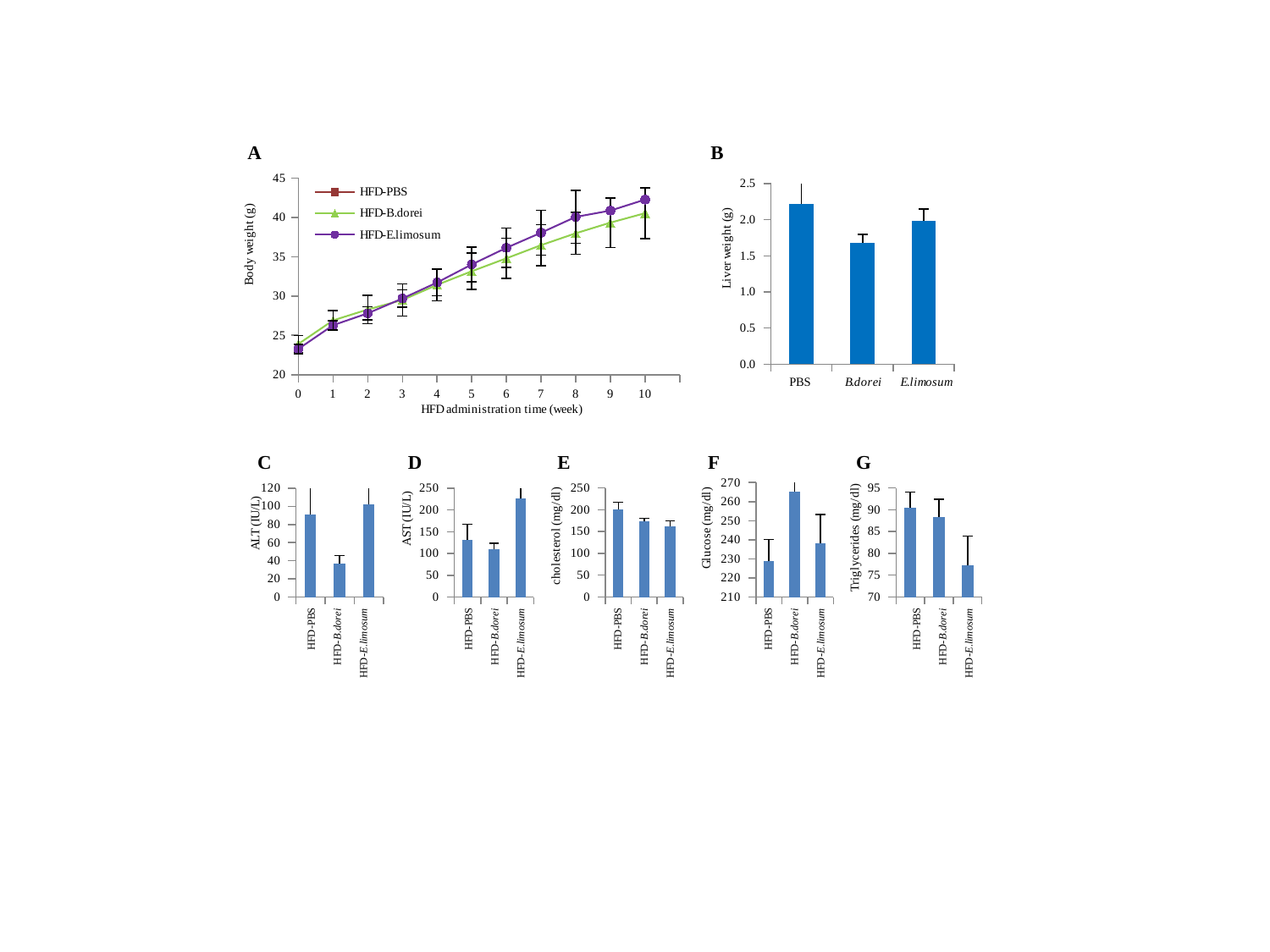

### Chart
| Category | HFD-PBS | HFD-B.dorei | HFD-E.limosum |
|---|---|---|---|
| 0 | 24.416666666666668 | 23.900000000000002 | 23.26666666666667 |
| 1 | 27.86666666666667 | 26.89999999999999 | 26.28333333333332 |
| 2 | 29.783333333333317 | 28.28333333333332 | 27.799999999999994 |
| 3 | 31.883333333333326 | 29.48333333333332 | 29.683333333333326 |
| 4 | 33.833333333333336 | 31.41666666666666 | 31.716666666666672 |
| 5 | 36.066666666666656 | 33.15 | 34.016666666666644 |
| 6 | 37.38333333333334 | 34.80000000000001 | 36.13333333333333 |
| 7 | 39.26666666666666 | 36.46666666666664 | 38.050000000000004 |
| 8 | 41.16666666666665 | 37.983333333333334 | 40.066666666666656 |
| 9 | 42.53333333333333 | 39.333333333333336 | 40.866666666666646 |
| 10 | 43.816666666666634 | 40.53333333333333 | 42.26666666666666 |
### Chart
| Category | |
|---|---|
| PBS | 2.2183333333333337 |
| B.dorei | 1.6833333333333338 |
| E.limosum | 1.9816666666666665 |
### Chart
| Category | |
|---|---|
| HFD-PBS | 91.33333333333329 |
| HFD-B.dorei | 36.5 |
| HFD-E.lomosum | 102.6 |
### Chart
| Category | GOT |
|---|---|
| HFD | 130.33333333333343 |
| HFD-B.dorei | 110.0 |
| HFD-E.lomosum | 226.0 |
### Chart
| Category | T-Chol |
|---|---|
| HFD | 201.33333333333343 |
| HFD-B.dorei | 173.16666666666657 |
| HFD-E.lomosum | 161.4 |
### Chart
| Category | Glucose |
|---|---|
| HFD | 228.66666666666657 |
| HFD-B.dorei | 265.1666666666669 |
| HFD-E.lomosum | 238.2 |
### Chart
| Category | TG |
|---|---|
| HFD | 90.5 |
| HFD-B.dorei | 88.33333333333329 |
| HFD-E.lomosum | 77.2 |
