# Supplementary material for: Bacterial metabolites directly modulate farnesoid X receptor activity
Source: Nutr Metab (Lond). 2015 Nov 24;12:48. doi: 10.1186/s12986-015-0045-y (PMC4657204; doi:10.1186/s12986-015-0045-y)
Supplement: Additional file 2: Figure S2. — Effects of FXR-stimulatory bacteria on intestinal and hepatic expression of FXR target genes. (A-F) mRNA levels in the ileum. (B) mRNA levels in the liver. mRNA levels were normalized to Gapdh mRNA levels via the relative standard curve method. Values are the mean ± SEM, n = 6. Differences from the PBS treatment group were calculated using Student’s t-test (*p < 0.05, **p < 0.01). (PPTX 108 kb) [file 12986_2015_45_MOESM2_ESM.pptx]

## Slide 1
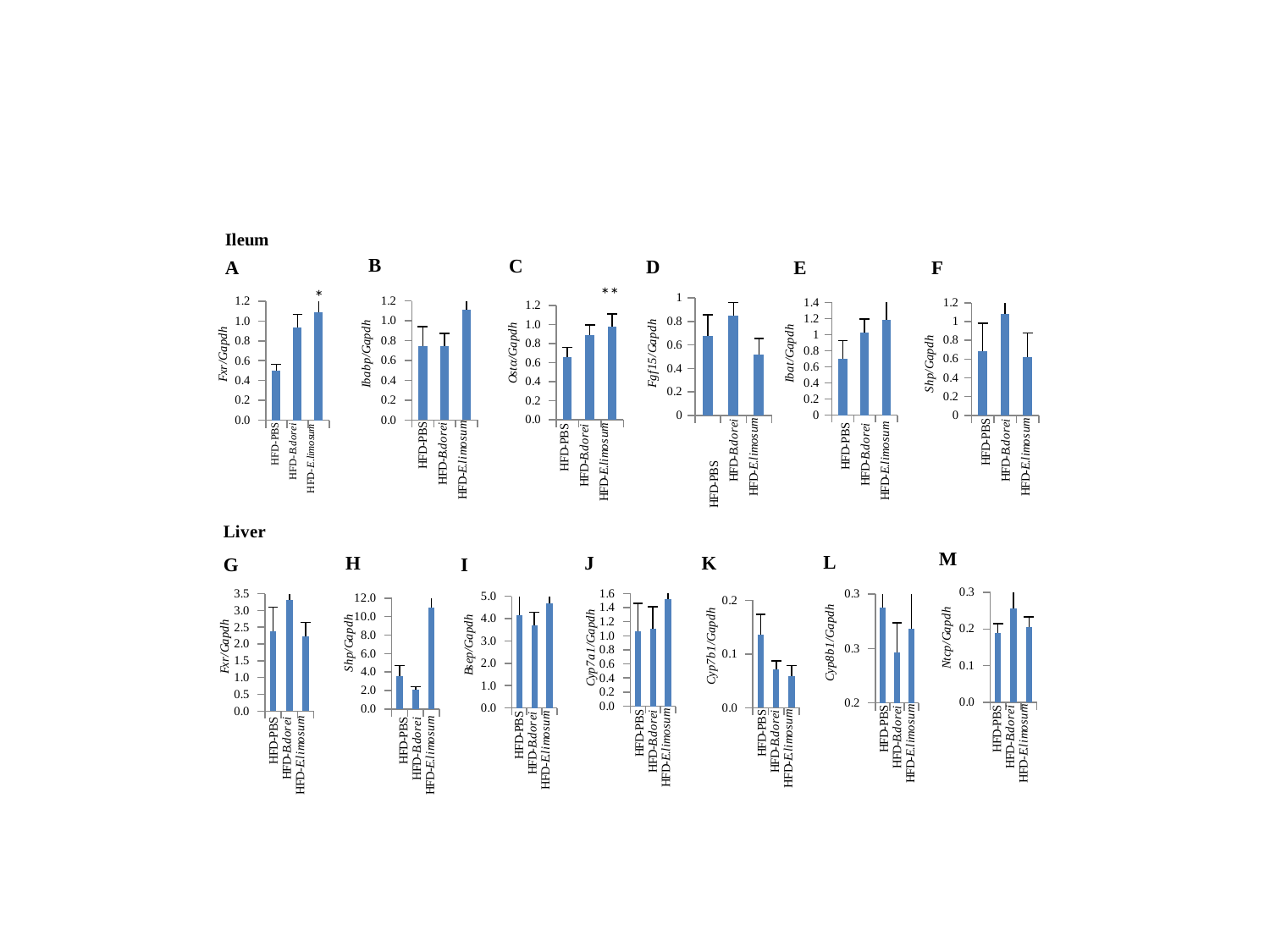

### Chart
| Category | |
|---|---|
| PBS | 0.6556204910592475 |
| B.dorei | 0.8891482296762016 |
| E.limosum | 0.978317426764256 |
### Chart
| Category | |
|---|---|
| PBS | 0.7046864696165636 |
| B.dorei | 1.0206365153162078 |
| E.limosum | 1.1825002695291122 |
### Chart
| Category | |
|---|---|
| PBS | 0.7405461814434489 |
| B.dorei | 0.7445173464160421 |
| E.limosum | 1.1143726880029978 |
### Chart
| Category | |
|---|---|
| PBS | 0.6796804502207968 |
| B.dorei | 1.0824782780152815 |
| E.limosum | 0.6213063545481584 |
### Chart
| Category | |
|---|---|
| PBS | 0.5031433791594607 |
| B.dorei | 0.9359068770044706 |
| E.limosum | 1.0861768312766653 |
### Chart
| Category | |
|---|---|
| PBS | 0.6743722647238473 |
| B.dorei | 0.8472623846371872 |
| E.limosum | 0.5184524607278572 |
### Chart
| Category | |
|---|---|
| PBS | 2.3867189097557167 |
| B.dorei | 3.304170362393387 |
| E.limosum | 2.220146407893808 |
### Chart
| Category | |
|---|---|
| PBS | 3.583661392609805 |
| B.dorei | 2.074128011066958 |
| E.limosum | 10.936161401498524 |
### Chart
| Category | |
|---|---|
| PBS | 1.061174114928373 |
| B.dorei | 1.0979584098847248 |
| E.limosum | 1.5174096635729812 |
### Chart
| Category | |
|---|---|
| PBS | 0.19014533762969832 |
| B.dorei | 0.25648864398140514 |
| E.limosum | 0.2048331371852087 |
### Chart
| Category | |
|---|---|
| PBS | 4.169461544715801 |
| B.dorei | 3.6950343491080635 |
| E.limosum | 4.667822745219843 |
### Chart
| Category | |
|---|---|
| PBS | 0.28767267952860814 |
| B.dorei | 0.24629044028537797 |
| E.limosum | 0.26793574857001473 |
### Chart
| Category | |
|---|---|
| PBS | 0.13557612234241742 |
| B.dorei | 0.07102968410515831 |
| E.limosum | 0.059380054941619624 |
